# Genome-Wide Exon-Capture Approach Identifies Genetic Variants of Norway Spruce Genes Associated With Susceptibility to *Heterobasidion parviporum* Infection

**DOI:** 10.3389/fpls.2018.00793

**Published:** 2018-06-12

**Authors:** Mukrimin Mukrimin, Andriy Kovalchuk, Leandro G. Neves, Emad H. A. Jaber, Matti Haapanen, Matias Kirst, Fred O. Asiegbu

**Affiliations:** ^1^Department of Forest Sciences, University of Helsinki, Helsinki, Finland; ^2^Department of Forestry, Faculty of Forestry, Hasanuddin University, Makassar, Indonesia; ^3^RAPiD Genomics, Gainesville, FL, United States; ^4^Natural Resources Institute Finland (LUKE), Helsinki, Finland; ^5^School of Forest Resources and Conservation, University of Florida, Gainesville, FL, United States

**Keywords:** exome resequencing, genome-wide association studies (GWAS), *Heterobasidion*, root and butt rot, Norway spruce, single-nucleotide polymorphism (SNP)

## Abstract

Root and butt rot caused by members of the *Heterobasidion annosum* species complex is the most economically important disease of conifer trees in boreal forests. Wood decay in the infected trees dramatically decreases their value and causes considerable losses to forest owners. Trees vary in their susceptibility to *Heterobasidion* infection, but the genetic determinants underlying the variation in the susceptibility are not well-understood. We performed the identification of Norway spruce genes associated with the resistance to *Heterobasidion parviporum* infection using genome-wide exon-capture approach. Sixty-four clonal Norway spruce lines were phenotyped, and their responses to *H. parviporum* inoculation were determined by lesion length measurements. Afterwards, the spruce lines were genotyped by targeted resequencing and identification of genetic variants (SNPs). Genome-wide association analysis identified 10 SNPs located within 8 genes as significantly associated with the larger necrotic lesions in response to *H. parviporum* inoculation. The genetic variants identified in our analysis are potential marker candidates for future screening programs aiming at the differentiation of disease-susceptible and resistant trees.

## Introduction

Norway spruce [*Picea abies* (L.) H. Karst.] is one of the dominant species in European boreal forests. As a major source of timber, it is one of the most commercially important species in Nordic countries. However, a combination of biotic and abiotic factors pose a threat to sustainable timber supply from Norway spruce plantations (Lindroth and St. Clair, 2013; Oliva et al., 2013; Robert et al., 2013; Lind et al., 2014; Wang et al., 2015). Among microbial pathogens of Norway spruce, necrotrophic fungi of the *Heterobasidion annosum* species complex occupy an outstanding position due to their devastating effect on commercial plantations (Keriö et al., 2014). Members of this species complex are causative agents of root and butt rot of conifer trees throughout the boreal and temperate zones of Northern hemisphere (Abu et al., 2004; Asiegbu et al., 2005a; Garbelotto and Gonthier, 2013; Gunulf et al., 2013; Skrøppa et al., 2015). They affect growth rate, mortality, and timber quality of conifer trees as well as steadily increase the risk of windfall (Gori et al., 2013; Keriö et al., 2014). The annual losses due to *Heterobasidion* infections are estimated at €50 million for Finnish forest industry (Gori et al., 2013; Piri and Hamberg, 2015), and at €790 million for European forest owners (Asiegbu et al., 2005a). Based on morphology, ecology, and molecular characters, *H. annosum* species complex is classified into five species, three of which occur in Europe: *H. annosum sensu stricto, H. parviporum*, and *H. abietinum* (Asiegbu et al., 2005a; Garbelotto and Gonthier, 2013; Hansson et al., 2014). Their preferred hosts are Scots pine, Norway spruce, and Silver fir, respectively (Asiegbu et al., 2005a). *H. parviporum* occurs primarily in forest areas where its major host, Norway spruce, grows naturally (Asiegbu et al., 2005a,b; Lind et al., 2014). The tree infection might occur either through wounds and damaged area on stem or, alternatively, pathogen might move from infected to healthy trees via root contacts (Asiegbu et al., 2005a).

Norway spruce trees possess a repertoire of defenses against microbial pathogens and pests. These defense reactions are commonly classified into physical and chemical defenses. An example of physical defense mechanisms is provided by thick outer bark, which acts as an efficient barrier that most pathogens cannot penetrate. It protects both dead tissues (heartwood) and living tissues (phloem, cambium and sapwood) (Kovalchuk et al., 2013). Chemical defense of spruce trees is achieved via production of diverse secondary metabolites such as flavonoids, lignans, phenolics, stilbenes, and terpenes. Many of them possess antimicrobial activities against potential pathogens, and they could delay or prevent the establishment of pathogens within the tree (Danielsson et al., 2011). Upon recognition of potential pathogens or tissue damage caused by microorganisms or insects, trees activate induced defenses that include cell wall reinforcement, production of phenolics, terpenes and pathogenesis-related (PR) proteins, and formation of traumatic resin ducts (TD), and polyphenolic parenchyma (PP) cells (Arnerup et al., 2013).

Growth rate and wood properties (Cunningham et al., 2006; Zubizarreta-Gerendiain et al., 2009; Luoranen and Viiri, 2016; Levkoev et al., 2017) and impact of climate change (Kolár et al., 2017) have been the major economic traits selected for in Norway spruce breeding programs. Recent studies have also aimed at identifying genes implicated in Norway spruce resistance against *H. parviporum* infection (Lind et al., 2014). Their results led to the identification of the gene *PaLAR3*, encoding leucoanthocyanidin reductase, an enzyme of the flavonoid biosynthetic pathway. A low frequency allele of this gene is associated with higher resistance to *H. parviporum* and increased content of (+)-catechin, a compound showing a fungistatic effect on *H. parviporum* (Nemesio Gorriz et al., 2016). However, Norway spruce resistance to the infection is a quantitative character, and it is likely to be controlled by multiple loci (Lind et al., 2014). Thus, further studies are required to identify additional genetic determinants of resistance against *H. parviporum* infection in Norway spruce.

The genome-based discoveries open the way to better understanding the effects of host genes associated with resistance to microbial pathogens. Availability of the draft sequence of Norway spruce genome (Nystedt et al., 2013) provides opportunities for high-throughput identification of genetic variants associated with the resistance to *H. parviporum* infection. Sequence capture or exon capture is a cost-efficient approach to determine genetic variation in species with a large genome size or without a reference genome having a wide range of scale of the capture from several targeted loci to a million target regions (Grover et al., 2012; Müller et al., 2015). This approach eliminates the need to set up and optimize thousands of PCR reactions for candidate genes, allowing instead for the parallel enrichment and sequencing of thousands of targeted regions. Exon capture has been successfully applied for such species as poplar, eucalyptus, polyploid wheat, polyploid switchgrass, and loblolly pine (Pavy et al., 2016). In the present study, we used the exon capture approach for the identification of Norway spruce genetic loci associated with the resistance or susceptibility to *H. parviporum* infection. The objectives of this study were:

1. to assess the genetic polymorphism among selected clonal lines of Norway spruce and
2. to identify genetic variants associated with the control of lesion lengths caused by *H. parviporum* inoculation.

## Materials and methods

### Plant and fungal material

The study material comprised 533 Norway spruce saplings. These represented 64 clonal lines which are a sample of the spruce breeding material for Central Finland (7 to 10 ramets per clonal line) (Table 1). The ortet trees were selected in two 10-year-old Norway spruce progeny trials in Pieksämäki (N 62°23′, E 27°17′) for their favorable phenotype (mainly for growth and late flushing). The ortets were propagated by rooted cuttings, which were then grown three seasons at the breeding nursery of the Natural Research Institute Finland. All the clones were untested and previously unexploited in research. Prior to the inoculation experiment, they were cultivated in a greenhouse (12 h daylight, day 22°C; night 18°C) at the Viikki campus of the University of Helsinki for 2 months.

**Table 1:** Detailed information on the studies clones.

**No**	**Clone**	**Parental trees**			**Number of ramets**	**Diameter (mm)**	**Height (cm)**	**Volume (cm^3^)**	**Geographical origin of mother**		**Degree days of mother**
		**Mother**	**Father**	**Origin**					**Latitude and longitude**	**Elevation (m)**	
1	V31002	K2467	Kun640	Pieksamäki	9	6.1	39.7	8.5	62°19′N, 027°17′E	140	1143
2	V31020	K3247	Kun640	Pieksamäki	9	5.3	33.8	5.5	62°19′N, 027°17′E	140	1126
3	V31055	E9801	+1296	Orivesi	9	6.5	36.2	9.1	61°42′N, 024°29′E	108	1195
4	V31062	K3238	Kun640	Pieksamäki	10	6.7	37.5	9.6	62°19′N, 027°17′E	140	1126
5	V31094	K3242	Kun640	Pieksamäki	8	5.4	21.8	3.7	62°19′N, 027°17′E	140	1126
6	V31159	E9792	+1297	Orivesi	9	6.4	34.4	8.0	61°36′N, 024°16′E	103	1240
7	V31163	K2468	Kun640	Pieksamäki	9	6.3	39.4	9.0	62°19′N, 027°17′E	140	1136
8	V31226	K2467	Kun640	Pieksamäki	8	9.0	52.1	23.7	62°19′N, 027°17′E	140	1143
9	V31278	K3234	Kun640	Pieksamäki	8	6.6	23.8	6.1	62°19′N, 027°17′E	140	1126
10	V31340	E9790	+1297	Orivesi	8	4.9	30.6	4.5	61°36′N, 024°16′E	103	1240
11	V31349	K2947	+1149	Leppävirta	8	5.2	31.3	5.3	62°39′N, 028°04′E	144	1176
12	V31355	K3247	Kun640	Pieksamäki	8	5.4	28.3	4.6	62°19′N, 027°17′E	140	1126
13	V31362	K2467	Kun640	Pieksamäki	9	7.9	55.2	16.0	62°19′N, 027°17′E	140	1143
14	V31390	K3247	Kun640	Pieksamäki	8	6.0	33.4	6.8	62°19′N, 027°17′E	140	1126
15	V31406	K3229	Kun640	Pieksamäki	7	5.6	18.1	3.2	62°19′N, 027°17′E	140	1126
16	V31417	E9797	+1296	Orivesi	9	7.3	39.9	13.5	61°42′N, 024°29′E	108	1195
17	V31418	E9797	+1296	Orivesi	8	7.3	42.4	12.4	61°42′N, 024°29′E	108	1195
18	V31460	K2468	Kun640	Pieksamäki	9	8.2	37.0	13.8	62°19′N, 027°17′E	140	1136
19	V31482	E9790	+1297	Orivesi	8	5.3	33.5	6.1	61°36′N, 024°16′E	103	1240
20	V31512	E9806	+1209	Jämsä	8	6.6	38.4	9.3	61°50′N, 024°47′E	145	1150
21	V31518	K9936	+1175	Mikkeli	9	6.6	34.0	8.2	61°49′N, 027°11′E	128	1149
22	V31521	E9812	+1209	Jämsä	8	6.0	23.0	4.9	61°50′N, 024°47′E	145	1150
23	V31523	K3284	+464	Multia	8	7.4	32.8	10.7	62°27′N, 024°52′E	161	1050
24	V31529	E9849	+1206	Jämsä	9	6.7	36.3	9.4	61°53′N, 025°19′E	216	1173
25	V31540	K3014	+1148	Leppävirta	7	6.7	32.6	7.8	62°24′N, 028°08′E	120	1176
26	V31552	E9925	+1175	Mikkeli	9	7.7	45.1	15.1	61°49′N, 027°11′E	128	1149
27	V31556	E9853	+1206	Jämsä	8	8.4	40.5	16.3	61°53′N, 025°19′E	216	1173
28	V31565	E9933	+1175	Mikkeli	8	7.7	40.1	13.6	61°49′N, 027°11′E	128	1149
29	V31570	K2984	+1146	Rautalampi	9	7.9	39.4	14.5	62°46′N, 026°45′E	98	1166
30	V31571	E9813	+1209	Jämsä	7	7.7	39.4	14.3	61°50′N, 024°47′E	145	1150
31	V31576	E9804	+1209	Jämsä	9	6.6	37.9	9.5	61°50′N, 024°47′E	145	1150
32	V31590	E9930	+1175	Mikkeli	9	7.8	43.2	14.7	61°49′N, 027°11′E	128	1149
33	V31608	E9918	+1175	Mikkeli	8	8.6	53.0	22.8	61°49′N, 027°11′E	128	1149
34	V31610	E9806	+1209	Jämsä	9	5.8	32.0	6.3	61°50′N, 024°47′E	145	1150
35	V31618	V4331	Koe19001	Pertunmaa (muurame)	8	6.3	34.5	7.9	61°25′N, 026°22′E	107	1109
36	V31634	K2988	+1146	Rautalampi	7	7.7	47.1	16.7	62°46′N, 026°45′E	98	1166
37	V31639	E9927	+1175	Mikkeli	8	8.3	42.6	17.2	61°49′N, 027°11′E	128	1149
38	V31644	E9806	+1209	Jämsä	9	7.4	39.0	12.3	61°50′N, 024°47′E	145	1150
39	V31645	E9806	+1209	Jämsä	9	8.0	42.4	15.2	61°50′N, 024°47′E	145	1150
40	V31650	V4324	Koe19001	Pertunmaa (muurame)	8	5.7	33.5	6.1	61°25′N, 026°22′E	107	1109
41	V31669	E9919	+1175	Mikkeli	9	6.3	37.7	8.8	61°49′N, 027°11′E	128	1149
42	V31675	E9810	+1209	Jämsä	8	7.4	34.3	10.9	61°50′N, 024°47′E	145	1150
43	V31680	K2983	+1146	Rautalampi	9	6.3	37.4	8.5	62°46′N, 026°45′E	98	1166
44	V31713	K2900	+1202	Vehmersalmi	7	7.4	31.6	10.0	62°42′N, 027°59′E	121	1158
45	V31714	E9930	+1175	Mikkeli	8	7.8	44.5	15.3	61°49′N, 027°11′E	128	1149
46	V31724	E9852	+1206	Jämsä	9	6.3	32.7	7.5	61°53′N, 025°19′E	216	1173
47	V31730	E9804	+1209	Jämsä	9	8.0	40.8	15.0	61°50′N, 024°47′E	145	1150
48	V31740	K2983	+1146	Rautalampi	8	5.4	33.5	5.8	62°46′N, 026°45′E	98	1166
49	V31742	E9850	+1206	Jämsä	9	6.8	33.8	8.9	61°53′N, 025°19′E	216	1173
50	V31753	K3284	+464	Multia	8	8.0	39.9	15.8	62°27′N, 024°52′E	161	1050
51	V31770	E9933	+1175	Mikkeli	8	8.0	48.3	17.4	61°49′N, 027°11′E	128	1149
52	V31795	E9804	+1209	Jämsä	8	5.1	34.0	5.3	61°50′N, 024°47′E	145	1150
53	V31809	E9921	+1175	Mikkeli	7	7.0	34.9	10.1	61°49′N, 027°11′E	128	1149
54	V31823	E9860	+1206	Jämsä	9	7.0	42.2	12.3	61°53′N, 025°19′E	216	1173
55	V31831	E9812	+1209	Jämsä	8	7.2	28.1	8.2	61°50′N, 024°47′E	145	1150
56	V31856	E9937	+1175	Mikkeli	7	7.2	40.6	11.7	61°49′N, 027°11′E	128	1149
57	V31858	K3009	+1147	Leppävirta	9	8.1	36.4	13.2	62°23′N, 028°15′E	120	1153
58	V31866	E9852	+1206	Jämsä	8	6.4	28.5	6.5	61°53′N, 025°19′E	216	1173
59	V31877	E9860	+1206	Jämsä	9	7.0	33.3	10.1	61°53′N, 025°19′E	216	1173
60	V31899	K2984	+1146	Rautalampi	8	7.1	37.6	10.8	62°46′N, 026°45′E	98	1166
61	V31905	E9804	+1209	Jämsä	9	7.7	36.1	12.6	61°50′N, 024°47′E	145	1150
62	V31939	E9851	+1206	Jämsä	8	7.3	32.3	10.8	61°53′N, 025°19′E	216	1173
63	V31969	E9860	+1206	Jämsä	8	6.2	37.9	8.1	61°53′N, 025°19′E	216	1173
64	V31998	K3284	+464	Multia	8	8.8	30.8	13.3	62°27′N, 024°52′E	161	1050

A *H. parviporum* isolate 04009 (a courtesy of Kari Korhonen, LUKE) was grown on 2% malt extract agar (MEA) plates supplemented with Norway spruce sawdust. The plates were inoculated with *H. parviporum* and incubated at 20°C for 4 weeks. For the mock inoculation, the sterile 2% MEA plates with 2% sawdust were used as a control.

### Phenotyping for susceptibility to *H. parviporum* infection

**Figure 1:**
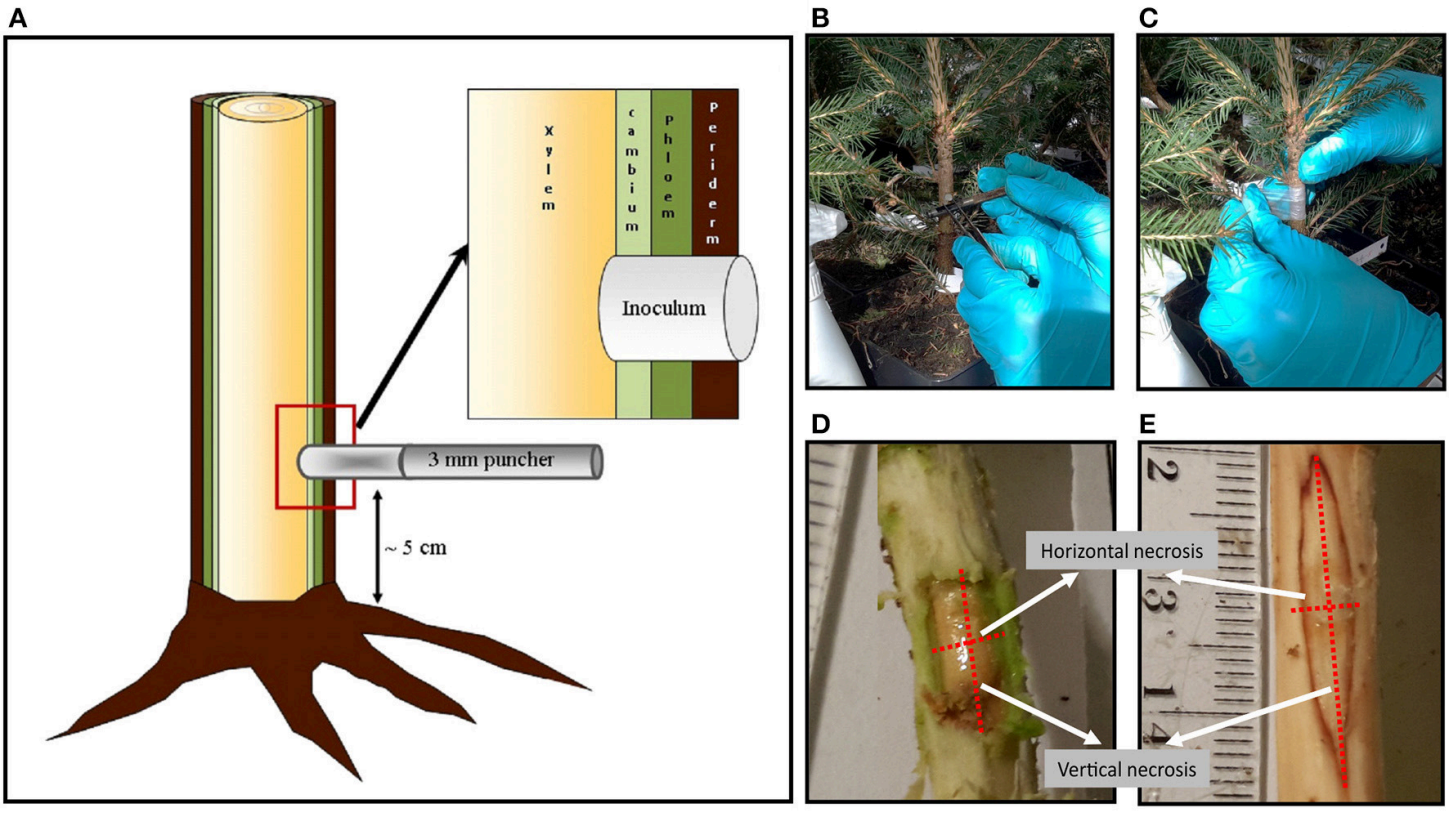
**(A)** illustration of inoculation method used in Norway spruce seedlings experiment. **(B)** Inoculated seedling stem with sawdust-agar pre-colonized by pathogen *H. parviporum* (nr. 04009). **(C)** Covering the inoculum with Parafilm. **(D)** Measurement horizontal and vertical necrosis on phloem **(E)** and xylem.

After the 2-month acclimatization, 4 to 7 saplings of each clone were inoculated with *H. parviporum*, and three saplings of each clone were mock-inoculated to perform as a control. The inoculation method used (Figure 1) was similar to the previously described one (Swedjemark and Stenlid, 1995, 1996; Sun et al., 2011; Keriö et al., 2014). The stem surface was sterilized with 70% ethanol before a hole was made through the bark with a 70% ethanol-sterilized puncher (3 mm diameter) to reach the xylem surface. The distance of the holes from the stem base was ~5 cm. Plugs of *H. parviporum*-colonized sawdust-containing malt extract agar of the same diameter as the holes were placed in the holes and then covered with Parafilm (Pechiney Plastic Packaging, Chicago, IL, USA). For control saplings, holes were inoculated with uncolonized sawdust-containing malt extract agar plugs. Saplings were harvested 2 months after inoculation. Harvested material was stored at −20°C until used. The necrotic lesions in phloem and xylem tissues were measured after removing the periderm tissues using a sterilized knife. The saplings dimensions (stem diameter, height, and volume) were also documented.

### Statistical analysis of phenotyping data

Data were analyzed using the SPSS version 25.0 (IBM Corporation, New York, USA) and Microsoft Excel 2016. A one-way analysis of variance (ANOVA) was performed to detect differences among the necrosis sizes of each clone and among the inoculation and mock treatments. The normality of the error variances was checked with Levene's test and by skewness and kurtosis values for the data distribution, the values for lesion size were converted to the logarithmic scale (Supplementary Files S1, S2). The effect of the clone on the lesion size was estimated with the following general linear model:

(1) $$\begin{array}{l} Y_{ij}=\mu +c_{i}+e_{ij} \end{array}$$

where *Y*_*ij*_ is the value of *ij*th observation, μ is the overall mean, *c*_*i*_ is the fixed effect of the clone i, and *e*_*ij*_ is the random residual term associated with the *i*th clone and *j*th ramet. Differences between clones were tested with the least significant difference (LSD) test between the means.

The growth rates of *H. parviporum* were assessed by *t*-tests for two-sample assuming unequal variances. Pearson's correlation analysis was used to determine correlations of diameter, height, and volume of the tested clonal lines with the sizes of necrotic lesions caused by *H. parviporum*. Differences were considered as statistically significant if *p*-value was below the threshold of 0.05.

### Estimation of heritability

Broad-sense heritability was estimated both for the growth traits (height, diameter, and volume of the saplings) and the lengths of various lesions using the following linear model:

(2) $$\begin{array}{l} {\text{y}}_{\text{ij}}=\mu +c_{\text{i}}+{\text{w}}_{\text{ij}} \end{array}$$

where y_ij_ is the ijth phenotypic observation of the trait y, μ is the general mean, c_i_ is the random effect ith clone and w_ij_ is the residual effect due to the jth sapling of the ith clone. The variance components of the model effects $\sigma_{\text{c}}^{2}$ and $\sigma_{\text{w}}^{2}$ were computed using procedure Mixed of the SAS package. Heritability was first calculated separately for both treatments (fungal vs. mock inoculation) as a ratio of the clonal variance to the phenotypic variance:

(3) $$\begin{array}{l} {\text{h}}^{2}={\text{r }\sigma^{2}}_{\text{c}}{\left(\sigma_{\text{c}}^{2}+\sigma_{\text{w}}^{2}\right)}^{-1} \end{array}$$

and its standard error as sqrt(Var ($\sigma_{\text{c}}^{2}$))/($\sigma_{\text{c}}^{2}$ + $\sigma_{\text{w}}^{2}$)^−1^.

Secondly, to analyze the magnitude of clone by treatment interaction in the lesion traits we used the mixed effect model:

(4) $$\begin{array}{l} {\text{y}}_{\text{ijk}}=\mu +{\text{c}}_{\text{i}}+{\text{T}}_{\text{j}}+{\text{c}}_{\text{i}}{\text{T}}_{\text{j}}+{\text{w}}_{\text{ijk}} \end{array}$$

where T_j_ is the fixed effect of the jth treatment (fungal inoculation or mock inoculation), and c_i_T_j_ is the random interaction effect between ith clone and jth treatment. This enabled us to separate the clone and clone by environment (treatment) interaction components of variance in the estimation of broad-sense heritability:

(5) $$\begin{array}{l} {\text{h}}^{2}=\sigma_{\text{c}}^{2}\;{\left(\sigma_{\text{c}}^{2}+\sigma_{\text{cT}}^{2}+\sigma_{\text{w}}^{2}\right)}^{-1} \end{array}$$

The relative magnitude of the interaction variance vs. the total genetic variance (R_cT_) was calculated as a simple ratio:

$$\begin{array}{l} {\text{R}}_{\text{cT}}=\sigma_{\text{cT}}^{2}{\left(\sigma_{\text{c}}^{2}+\sigma_{\text{cT}}^{2}\right)}^{-1} \end{array}$$

### Library preparation and target enrichment sequencing

Total genomic DNA was extracted from the needles using a standard cetyl–trimethyl ammonium bromide (CTAB) method with modifications described in Terhonen et al. (2011). DNA concentrations were estimated with PicoGreen dsDNA quantification assay (ThermoFisher Scientific, USA) and DNA integrity was analyzed by visualizing the DNA on a 0.8% w/v agarose electrophoresis gel. A total of 500 ng was utilized for construction of libraries compatible with Illumina sequencing machines. In summary, the DNA was mechanically sheared to a mean fragment size of 300 bp, followed by repair of the ends of the molecules, phosphorylation and adenylation. Common adapters suited for Illumina sequencing were ligated on each side of the molecules containing 8 bp indexes (i5 and i7). The ligated libraries were amplified with 10 cycles of PCR with common primers to enrich the libraries for properly ligated molecules and the resulting libraries were quantified with PicoGreen. A set of 80,000 probes was utilized to capture genic regions of the Norway spruce genome (Vidalis et al., in review). In summary, these probes target 34761 genes with a median of two probes per gene, and were selected based on their uniqueness on the genome. Probe sequences were designed based on the *P. abies* v1.0 genome (Nystedt et al., 2013) and RNA-based, 120 nucleotide long probes were synthesized by Agilent Technologies. Sixteen libraries were equimolarly combined to a total of 750 ng for target enrichment, which was performed following SureSelect Target Enrichment System (Agilent Technologies). Enriched libraries were sequenced using Illumina HiSeq3000 instrument on a 2 × 100 bp sequencing mode. The library construction and enrichment sequencing were performed by RAPiD Genomics (USA). The generated sequence reads were submitted to the GenBank Short Reads Archive (SRA) and are available under the BioProject accession number PRJNA450911.

### Variant identification and genome-wide association analysis

The generated sequencing data was processed to identify the SNPs segregating in the population. Short reads were filtered for quality and aligned to the scaffolds that contain probes of the *P. abies* v1.0 genome using Mosaik version 2.1 as described before (Neves et al., 2013). The resulting alignment BAM files were used to identify SNPs with Freebayes v1.0.2 (Garrison and Marth, 2012) on a populational level (parameters: –max-complex-gap 1 –theta 0.01 –no-complex –no-mnps –no-indels). The resulting SNPs were filtered using VCF.filter v0.1.13 (Müller et al., 2017) to keep markers with a minimum quality of 10, an average sequencing depth between 15 and 150, a minimum allele frequency of 0.01, a missing data lower than 0.4 and assigning genotypes with sequencing depth lower than 3 as missing. The VCF file containing the information on the identified SNPs was deposited to the Dryad repository (Mukrimin et al., 2018).

The filtered markers for the 64 clonal lines were subsequently used for genome-wide association analysis (GWAS). The average xylem lesion size for each clone was used as a quantitative phenotype for the GWAS, which was carried out using EMMAX (Kang et al., 2010) including the BN kinship matrix in the model to control for relatedness in the population. To control for multiple testing, the Bonferroni and the Benjamini and Hochberg (1995) procedures were used (α = 0.05). The Manhattan and Quantile-Quantile plot (Q-Q plot) were calculated using the qqman package on R (Turner SD. Qqman: an R Package For Visualizing Gwas Results Using Q-Q And Manhattan Plots. 2014; https://doi.org/10.1101/005165). Since the spruce genome is not ordered and fragmented, the *p*-values plotted on the Manhattan plot do not represent any physical or genetic distance, but rather each SNP was simply considered a coordinate on the x-axis for visualization purposes (Supplementary File S3).

## Results

### Variation in lesion length in response to fungal inoculation

In our experiment, the lesions formed in response to inoculation with *H. parviporum* were significantly larger than the lesions developing after mock treatment. The differences between mock and fungal inoculation treatments were observed both in xylem and in phloem, and they were statistically significant (*p* < 0.001) in both cases (Table 2).

**Table 2:** Size of necrotic lesions in spruce saplings (mean ± SE) for inoculation and mock treatments.

**Tissues and treatment**	**Sample size**	**Vertical necrosis (mm)**	**Horizontal Necrosis (mm)**
**PHLOEM**			
Inoculation	341	8.3 ± 0.2	6.1 ± 0.1
Mock treatment	192	4.0 ± 0.1	2.1 ± 0.1
**XYLEM**			
Inoculation	341	8.6 ± 0.2	3.2 ± 0.1
Mock treatment	192	4.6 ± 0.1	2.0 ± 0.1

The lesions developed faster in vertical than in horizontal direction, as indicated by significant differences between vertical and horizontal lesion lengths in all treatments [e.g., *F*_(1, 680)_ = 96.58, *F*_(1, 680)_ = 547.82, *F*_(1, 380)_ = 130.52, and *F*_(1, 381)_ = 402, respectively at *p* < 0.001]. Horizontal lesion length in phloem was significantly higher than in xylem (*p* < 0.001), but there was no significant differences between the tissues in the vertical lesion length.

**Figure 2:**
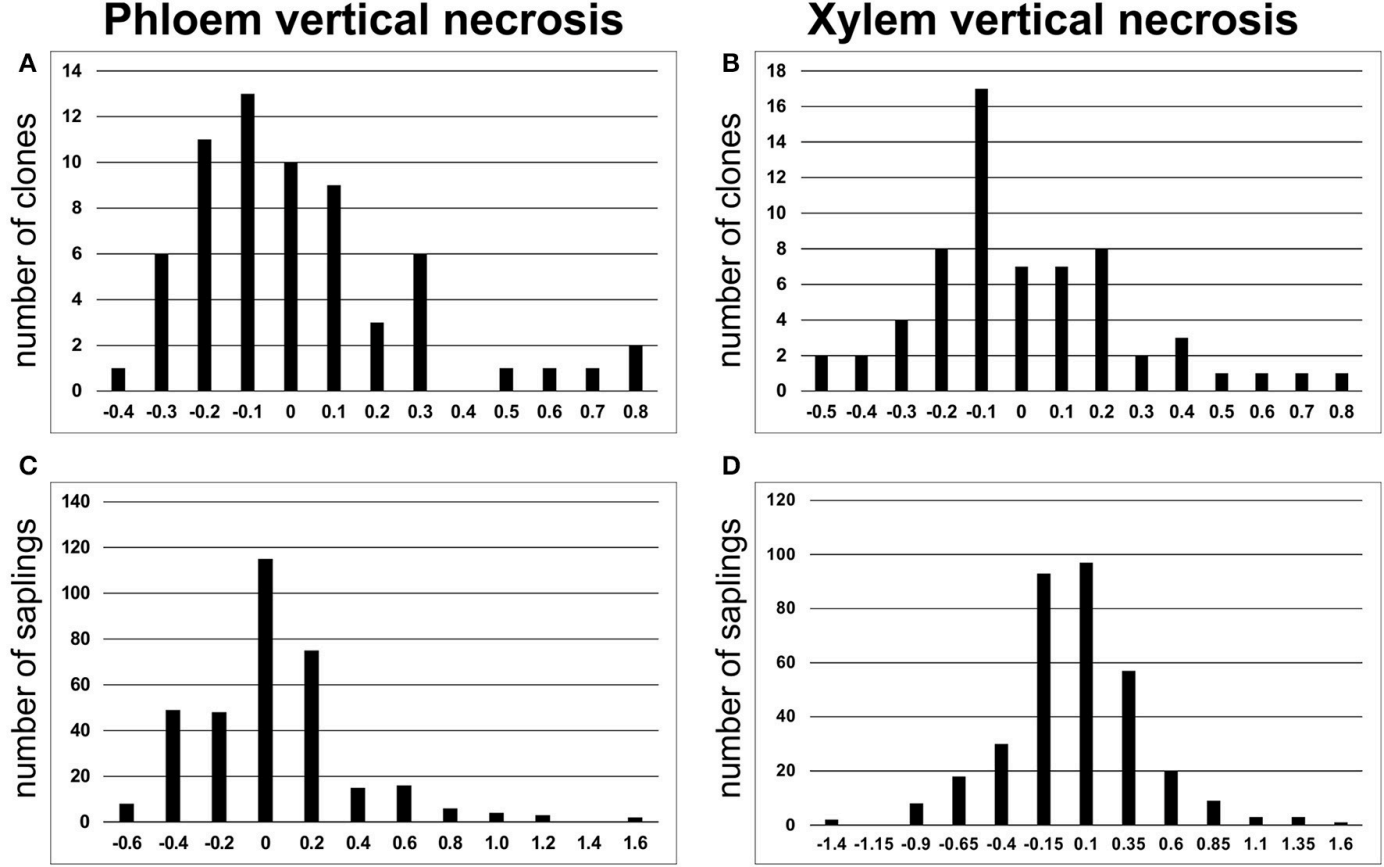
LogNormal distributions of vertical necrotic stem lesion size in phloem **(A, C)** and xylem **(B, D)** based on number of clones and individual saplings, respectively.

**Figure 3:**
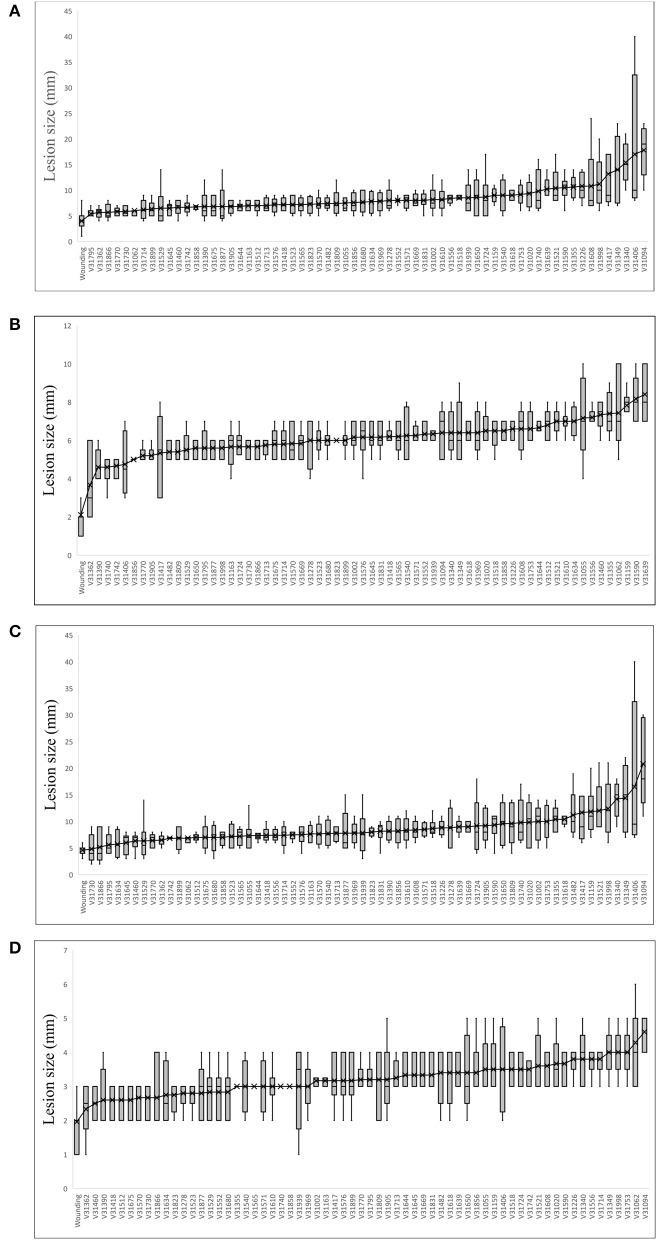
Clone means of lesion size in **(A)** phloem vertical necrosis; **(B)** phloem horizontal necrosis; **(C)** xylem vertical necrosis; and **(D)** xylem horizontal necrosis.

The analyzed spruce clones displayed significant differences in size of necrotic lesions both in phloem and in xylem [*F*_(63, 277)_ = 2.84 and *F*_(63, 277)_ = 2.77, respectively at *p* < 0.001]. The distribution of lesion length among the analyzed clones was similar to normal (Figure 2). The data for the individual clones are summarized in Figure 3. Clonal lines V31094 and V31406 had the largest lesion sizes and were classified as the most susceptible to the infection.

Based on Pearson correlation coefficients (r), there were significant negative correlations between the growth traits (stem diameter, seedling height, and volume) and the observed lesion sizes. The strongest negative correlation was found in inoculated xylem that differed significantly at *p* < 0.01 (Table 3).

**Table 3:** Pearson correlation coefficients (r) according to both phloem and xylem lesion size to growth traits.

**Necrosis type**	**Diameter**	**Height**	**Volume**
**PHLOEM**			
**Inoculation**			
Vertical lesion	−0.168^**^	−0.189^**^	−0.127^*^
Horizontal lesion	0.188^**^	0.074	0.172^**^
**Mock treatment**			
Vertical lesion	−0.244^**^	−0.339^**^	−0.279^**^
Horizontal lesion	−0.167^*^	−0.113	−0.132
**XYLEM**			
**Inoculation**			
Vertical lesion	−0.311^**^	−0.323^**^	−0.292^**^
Horizontal lesion	−0.107^*^	−0.064	−0.073
**Mock Treatment**			
Vertical lesion	−0.295^**^	−0.338^**^	−0.322^**^
Horizontal lesion	−0.06	−0.015	−0.013

Diameter measured 5 cm from the stem's base Significant correlation at

* p < 0.05 and

** *p < 0.01*.

The heritability estimates, indicating the proportion of clonal variation of the total phenotypic variation in each treatment, were moderate to large, ranging from 0.18 to 0.80. The levels of heritability were consistently larger for the clones subjected to mock treatment than the respective estimates for inoculated trees. The genetic coefficients of variation displayed the similar tendency (Table 4). The clonal variance used in calculating these heritability values was in fact composed of two components of variation: the true genetic (clonal) and genotype by treatment interaction variances. When these two sources of variation were distinguished by Equation (4), much smaller heritability estimates were obtained both for the lesion traits (0.15–0.23) and the growth traits (0.07–0.13) (Table 5). The relative magnitude of the genotype by treatment interaction variance (Equation 5) is generally low, but markedly high for phloem horizontal necrosis.

**Table 4:** Estimates of among-clone and within-clone (residual) variance components and their standard errors.

**Trait**	**Treatment**	**σ_clone_ ± std_err**	**σ_residual_ ± std_err**	**${\text{h}}_{\text{clone}}^{2}$ ± std_err^a^**	**GCV, %^a^**	**Mean**
Diameter (mm)	Inoculation	0.73 ± 0.20	1.82 ± 0.16	0.29 ± 0.08	12.3	7.0
Diameter (mm)	Mock treatment	0.94 ± 0.30	2.01 ± 0.25	0.32 ± 0.10	14.1	6.9
Height (dm)	Inoculation	0.40 ± 0.08	0.25 ± 0.02	0.62 ± 0.12	17.1	3.7
Height (dm)	Mock treatment	0.56 ± 0.11	0.21 ± 0.03	0.73 ± 0.15	20.6	3.7
Phloem horizontal necrosis (mm)	Inoculation	0.56 ± 0.14	1.04 ± 0.09	0.35 ± 0.08	12.2	6.1
Phloem horizontal necrosis (mm)	Mock treatment	2.73 ± 0.53	0.67 ± 0.08	0.80 ± 0.16	77.7	2.1
Phloem vertical necrosis (mm)	Inoculation	4.09 ± 1.17	11.61 ± 1.00	0.26 ± 0.07	24.3	8.3
Phloem vertical necrosis (mm)	Mock treatment	0.92 ± 0.23	1.03 ± 0.13	0.47 ± 0.12	24.0	4.0
Volume (cm^3^)	Inoculation	14.26 ± 3.48	25.08 ± 2.16	0.36 ± 0.09	34.9	10.8
Volume (cm^3^)	Mock treatment	23.66 ± 5.98	27.05 ± 3.41	0.47 ± 0.12	45.7	10.6
Xylem horizontal necrosis (mm)	Inoculation	0.11 ± 0.04	0.53 ± 0.05	0.18 ± 0.06	10.5	3.2
Xylem horizontal necrosis (mm)	Mock treatment	0.33 ± 0.08	0.30 ± 0.04	0.53 ± 0.12	29.4	2.0
Xylem vertical necrosis (mm)	Inoculation	4.53 ± 1.31	13.37 ± 1.15	0.25 ± 0.07	24.7	8.6
Xylem vertical necrosis (mm)	Mock treatment	0.91 ± 0.28	1.83 ± 0.23	0.33 ± 0.10	20.8	4.6

a *$h_{clone}^{2}$ is the clonal heritability and GCV is the genetic coefficient of variation (square-root of the clonal variance component divided by the mean)*.

**Table 5:** Clone, clone by treatment and residual variance components, broad-sense heritability (h2) and the relative magnitude of the clone by treatment variance (RcT).

**Trait**	**σclone**	**σclone by trt**	**σresidual**	**σTotal**	**h2**	**seh2**	**RcT**
Phloem vertical necrosis, mm	1,96	0,61	8,03	10,60	0,18	0,07	0,24
Phloem horizontal necrosis, mm	0,37	1,16	0,93	2,45	0,15	0,09	0,76
Xylem vertical necrosis, mm	2,79	0,14	9,43	12,37	0,23	0,07	0,05
Xylem horizontal necrosis, mm	0,13	0,05	0,47	0,65	0,21	0,06	0,27
Diameter	0,78	0,03	1,89	2,70	0,29	0,07	0,03
Height	0,45	0,01	0,24	0,70	0,65	0,13	0,02
Volume	17,62	0,00	25,85	43,47	0,41	0,09	0,00

### Sequence capture and sequencing of gene space in norway spruce

We genotyped 64 clonal lines of Norway spruce by resequencing of predicted exons corresponding to 34 761 gene models. On average, 6.3 million reads were generated per clonal line. Reads were aligned to Norway spruce reference genome, with the median experiment-wide sequencing depth of 40.35×. The efficiency of the sequence enrichment strategy was high, capturing 79 170 out of 80 000 probes. Calling of genetic variants identified 373 384 high-quality biallelic SNPs.

### Identification of sequence variants associated with the necrotic lesion size

Genome-wide association analysis identified 34 genes (36 SNPs) that were significantly associated (at 5% FDR significance level) with the size of necrotic lesions formed in response to fungal inoculation (Supplementary File S4). As we had a limited number of clonal lines available for GWAS analysis, we additionally selected only those variants, which occurred in at least 4 clonal lines. The resulting set included 10 SNPs located within 8 gene models (Table 6). All of the identified variants were associated with larger necrotic lesions and belonged to low frequency alleles (frequency among studied clonal lines <5%). Three of the identified genes were of particular interest because of their potential involvement in the regulation of plant defense responses: the gene MA_940838g0010 with a similarity to *Arabidospis thaliana* ILITYHIA, which is required for the establishment of systemic acquired resistance (Monaghan and Li, 2010), the gene MA_4047g0010 encoding a predicted subtilase (subtilisin-like serine peptidase), and the gene MA_3905g0010 with a similarity to the plant-specific class of HD2-type histone deacetylases.

**Table 6:** SNPs identified by GWAS as significantly associated with the size of necrotic lesions formed in response to fungal inoculation.

**Contig**	**Pos**	**Beta**	**BONF**	**MAF**	**Lines with the variant allele**	**Gene model**	**Exon/intron**	**Predicted function**	**Nucleotide variant 0**	**Nucleotide variant 1**	**Effect**
MA_10430455	20871	−5.22	1.3E-03	0.042	4	MA_10430455g0010	Intron	Apolipoprotein	C	A	n/a
MA_10435574	26634	−5.00	1.7E-02	0.042	4	MA_10435574g0010	Intron	Similar to A. thaliana SIGNAL PEPTIDE PEPTIDASE-LIKE 1	A	T	n/a
MA_10437232	6258	−5.12	6.0E-03	0.042	4	MA_10437232g0010	Exon	Similar to A. thaliana TCS1 (TRICHOME CELL SHAPE 1)	G	C	No effect; Thr (ACC -> ACG) (coding sequence is located on complementary strand)
MA_10437232	6283	−5.12	6.0E-03	0.042	4	MA_10437232g0010	Exon	Similar to A. thaliana TCS1 (TRICHOME CELL SHAPE 1)	C	A	Gly (GGG) -> Val (GUG) (coding sequence is located on complementary strand)
MA_20554	10808	−5.50	2.6E-02	0.047	6	MA_20554g0010	Intron	Small nuclear ribonucleoprotein	G	A	n/a
MA_3905	29322	−7.24	1.2E-05	0.032	4	MA_3905g0010	Intron	Putative HD2-type histone deacetylase	C	T	n/a
MA_3905	29323	−7.24	1.2E-05	0.032	4	MA_3905g0010	Intron	Putative HD2-type histone deacetylase	T	G	n/a
MA_4047	19725	−4.74	2.1E-03	0.049	5	MA_4047g0010	Exon	Subtilase (subtilisin-like serine protease)	T	C	No effect; Ser (AGU -> AGC)
MA_479900	6145	−5.48	6.9E-05	0.041	4	MA_479900g0010	Exon	Uncharacterized F-box containing protein	G	A	Glu (GAG) -> Lys (AAG)
MA_940838	2433	−6.54	1.7E-04	0.042	5	MA_940838g0010	Exon	ILITHYIA. A. thaliana HEAT repeat protein involved in immunity	C	A	Ser (UCU) -> Tyr (UAU)

## Discussion

In this study, we characterized a set of Norway spruce clonal lines to explore the genetic control of susceptibility to *H. parviporum* infection. As an indicator of susceptibility, we used size of necrotic lesions developing in response to fungal inoculation. The artificial fungal inoculation of seedlings and saplings has been widely practiced under controlled greenhouse conditions to investigate tree susceptibility to *Heterobasidion* infection (Swedjemark and Stenlid, 1996; Swedjemark et al., 2001; Sun et al., 2011; Krokene et al., 2012). This approach is much faster than the field experiments (Swedjemark and Stenlid, 1996), but yet much more complex than a potential selection via genetic markers. Inoculation with *Heterobasidion* results in the pathogen spread in host tissues and in the development of necrotic lesions around the inoculation point.

In this study, the necrotic lesions significantly differed among the tested clones, which concurs with the results of previous studies (Swedjemark and Stenlid, 1996; Swedjemark et al., 2001; Keriö et al., 2014; Skrøppa et al., 2015). Our findings show that saplings with lower values of growth parameters (stem diameter, height, and volume) appeared considerably more sensitive to fungal inoculation and displayed larger necrotic lesions in response to *H. parviporum* infection. This is in accordance with previous results that reported a strong negative correlation between growth traits and *H. parviporum* resistance (Karlsson et al., 2008). The investigated clones represented a practically random sample of genotypes from a single breeding population aimed at a specific region (Central Finland). We could not detect any common variables characterizing these clones that would explain the differences observed in their susceptibility.

Heritability is often used in quantitative genetics, behavior genetics, selective breeding, among other fields to quantify the ratio of genetic to phenotypic variation in a specific population and a specific environment. In this study, all the traits showed moderate to high heritability, suggesting a relatively small role of environmental differences in the trait expression when the analyses were performed separately for the two treatments. Interestingly, heritability estimates for traits measured after the mock treatment were consistently higher than the respective estimates on clones subjected to inoculation, suggesting these traits could be indeed considered to be genetically different. In the second analysis for the combined data (including a fixed treatment effect and a random clone by treatment variance) all the traits studied showed moderate heritability. The relative magnitude of the interaction was high for a single trait, phloem horizontal necrosis, but small to moderate for the rest of the lesion traits and very small for all of the three growth traits. This kind of discrepancy is interesting, yet difficult to explain. The finding needs to be verified in further studies to make sure that it was not just a random occurrence.

GWAS became an important tool in the identification of genetic loci controlling economically important traits in various plant species (Kump et al., 2011; Tian et al., 2011; Li et al., 2013; Wei et al., 2015). Our analysis identified 10 SNPs associated with the control of the size of necrotic lesions developed in response to *H. parviporum* inoculation. These SNPs correspond to 8 genes. None of the identified genes was previously reported as being implicated in control of resistance of Norway spruce to *Heterobasidion* infection. At the same time, none of the SNP markers associated with the Norway spruce resistance to *H. parviporum* in a previous screening program (Lind et al., 2014) was recovered in our analysis. Possible explanations for the lack of overlap of our results with the previous ones could be differences in the employed marker identification strategies; the populations used, whereas in the case of our work a multi-pedigree breeding population was used vs. a single bi-parental QTL cross used in the referenced study; and the relatively small number of samples employed in both studies.

Our analysis identified several genes, which might be involved in the regulation of plant defense responses in Norway spruce. The identification of a gene showing similarity to *A. thaliana* ILITYHIA (ILA) in our study is particularly noteworthy, as the corresponding protein was demonstrated to play a key role in the induced defense reactions in *Arabidopsis* (Monaghan and Li, 2010). Furthermore, by controlling chloroplast development, it might be implicated in the regulation of the ROS accumulation and programmed cell death in response to a pathogen attack (Faus et al., 2018). However, nothing is known about the function of this protein in other plant species, including conifer trees. The role of this gene in defense reactions of Norway spruce against *H. parviporum* infection deserves further studies.

The gene MA_4047g0010 encodes a predicted subtilase (subtilisin-like serine peptidase). This protein family is largely expanded in plant genomes, and their physiological functions include the control of growth and development, regulation of programmed cell death and senescence and plant responses to biotic and abiotic stressors (Schaller et al., 2018). Several plant subtilases are important modulators of plant immune responses (Tornero et al., 1996; Ramírez et al., 2013; Serrano et al., 2016). A specific group of subtilases, called phytaspases, are involved in the regulation of programmed cell death (hypersensitive response) triggered by the pathogen attack (Chichkova et al., 2004; Schaller et al., 2018). Our transcriptomic profiling of asymptomatic and symptomatic Norway spruce trees naturally infected by *Heterobasidion* sp. showed that the gene MA_4047g0010 had higher expression level in symptomatic trees, with the *p* value (*p* = 0.06) close to the significance threshold (Supplementary File S5). However, taking into account a large number of the predicted subtilase genes in spruce genome, it is difficult to predict the specific role of the identified gene, and its characterization would require further experimental work.

The MA_3905g0010, identified in our analysis, shows similarity to the plant-specific class of HD2 histone deacetylases. Members of this class play a role, among others, in ABA and abiotic stress responses (Sridha and Wu, 2006; Luo et al., 2012; Han et al., 2016). They might potentially contribute to the ABA-mediated regulation of programmed cell death, which, in turn, might be one of the factors determining sensitivity or resistance to necrotrophic pathogens (Coll et al., 2011).

The connection of the remaining genes with the responses of spruce trees to fungal infection are less clear. No obvious link between their deduced function and the response of spruce trees against fungal infection could be established. However, it is possible that identified markers are physically linked with neighboring genes, located in the adjacent chromosomal loci. Unfortunately, the draft genome assembly of Norway spruce is unordered and highly fragmented (N50 = 4,869 bp; Nystedt et al., 2013), which makes it not always possible to find genes located adjacent to the identified SNP markers.

The identified genetic variants represent low frequency alleles (occurrence frequency among analyzed clonal lines <5%). This observation indicates that more extensive genotyping programs involving several hundreds of individuals might be required to identify the most relevant alleles controlling responses of Norway spruce to *H. parviporum* infection. In parallel, genomic selection approaches can be used to advance the selection of tolerant trees in the breeding program, as these methods do not rely on the dissection of quantitative traits to rank the most favorable trees.

This study is the first attempt to identify genes involved in the control of Norway spruce resistance to *H. annosum* infection using sequence capture enrichment strategy. Obtained results illustrate the suitability of this approach for genotyping of Norway spruce, a species with a very large genome and a high content of repetitive elements. Some of the identified genes are promising candidates, and further analysis should investigate their role in spruce defense reactions. However, our study also illustrates that even larger efforts might be required to identify genetic variants controlling economically important traits and occurring in low frequency in natural populations.

## Author contributions

Phenotyping of Norway spruce saplings was performed by MM and EJ; total genomic DNA extraction was done by MM and EJ; genetic and genome-wide association analyses were done by LN; heritability analysis was carried out by MH; MM, AK, and LN analyzed the data; MM and AK prepared the manuscript draft; MK and FA conceived the study and contributed to the experiment design. All authors read and approved the final version of the manuscript.

### Conflict of interest statement

The authors declare that the research was conducted in the absence of any commercial or financial relationships that could be construed as a potential conflict of interest.

## References

[B1] Abu S. M. Li G. Asiegbu F. O. (2004). Identification of *Heterobasidion annosum* (S-type) genes expressed during initial stages of conidiospore germination and under varying culture conditions. FEMS Microbiol. Lett. 233, 205–213. 10.1016/j.femsle.2004.02.011 15063488

[B2] Arnerup J. Nemesio-Gorriz M. Lundén K. Asiegbu F. O. Stenlid J. Elfstrand M. (2013). The primary module in Norway spruce defence signalling against *H. annosum* s.l. seems to be jasmonate-mediated signalling without antagonism of salicylate-mediated signalling. Planta 237, 1037–1045. 10.1007/s00425-012-1822-8 23223898

[B3] Asiegbu F. O. Adomas A. Stenlid J. (2005a). Conifer root and butt rot caused by *Heterobasidion annosum* (Fr.) Bref. s.l. Mol. Plant Pathol. 6, 395–409. 10.1111/j.1364-3703.2005.00295.x 20565666

[B4] Asiegbu F. O. Nahalkova J. Li G. (2005b). Pathogen-inducible cDNAs from the interaction of the root rot fungus *Heterobasidion annosum* with Scots pine (*Pinus sylvestris* L.). Plant Sci. 168, 365–372. 10.1016/j.plantsci.2004.08.010

[B5] Benjamini Y. Hochberg Y. (1995). Controlling the false discovery rate - a practical and powerful approach to multiple testing. J. R. Stat. Soc. Ser. B 57, 289–300.

[B6] Chichkova N. V. Kim S. H. Titova E. S. Kalkum M. Morozov V. S. Rubtsov Y. P.. (2004). A plant caspase-like protease activated during the hypersensitive response. Plant Cell 16, 157–171. 10.1105/tpc.017889 14660804 PMC301402

[B7] Coll N. S. Epple P. Dangl J. L. (2011). Programmed cell death in the plant immune system. Cell Death Differ. 18:1247. 10.1038/cdd.2011.37 21475301 PMC3172094

[B8] Cunningham C. Zimmermann N. E. Stoeckli V. Bugmann H. (2006). Growth response of Norway spruce saplings in two forest gaps in the Swiss Alps to artificial browsing, infection with black snow mold, and competition by ground vegetation. Can. J. For. Res. 36, 2782–2793. 10.1139/x06-156

[B9] Danielsson M. Lundén K. Elfstrand M. Hu J. Zhao T. Arnerup J.. (2011). Chemical and transcriptional responses of Norway spruce genotypes with different susceptibility to Heterobasidion spp. infection. BMC Plant Biol. 11:154. 10.1186/1471-2229-11-154 22067529 PMC3240162

[B10] Faus I. Niñoles R. Kesari V. Llabata P. Tam E. Nebauer S. G. (2018). Arabidopsis ILITHYIA protein is necessary for proper chloroplast biogenesis and root development independent of eIF2α phosphorylation. J. Plant Physiol. 224–225, 173–182. 10.1016/j.jplph.2018.04.003 29680783

[B11] Garbelotto M. Gonthier P. (2013). Biology, epidemiology, and control of Heterobasidion species worldwide. Annu. Rev. Phytopathol. 51, 39–59. 10.1146/annurev-phyto-082712-102225 23642002

[B12] Garrison E. Marth G. (2012). Haplotype-based variant detection from short-read sequencing. arXiv preprint arXiv:1207.3907.

[B13] Gori Y. Cherubini P. Camin F. La Porta N. (2013). Fungal root pathogen (*Heterobasidion parviporum*) increases drought stress in Norway spruce stand at low elevation in the Alps. Eur. J. For. Res. 132, 607–619. 10.1007/s10342-013-0698-x

[B14] Grover C. E. Salmon A. Wendel J. F. (2012). Targeted sequence capture as a powerful tool for evolutionary analysis. Am. J. Bot. 99, 312–319. 10.3732/ajb.1100323 22268225

[B15] Gunulf A. Wang L. Englund J. E. Rönnberg J. (2013). Secondary spread of *Heterobasidion parviporum* from small Norway spruce stumps to adjacent trees. For. Ecol. Manage. 287, 1–8. 10.1016/j.foreco.2012.09.011

[B16] Han Z. Yu H. Zhao Z. Hunter D. Luo X. Duan J.. (2016). *AtHD2D* gene plays a role in plant growth, development, and response to abiotic stresses in *Arabidopsis thaliana*. Front. Plant Sci. 7:310. 10.3389/fpls.2016.00310 27066015 PMC4815178

[B17] Hansson D. Wubshet S. Olson Å. Karlsson M. Staerk D. Broberg A. (2014). Secondary metabolite comparison of the species within the *Heterobasidion annosum* s.l. complex. Phytochemistry 108, 243–251. 10.1016/j.phytochem.2014.08.028 25260338

[B18] Kang H. M. Sul J. H. Service S. K. Zaitlen N. A. Kong S. Y. Freimer N. B.. (2010). Variance component model to account for sample structure in genome-wide association studies. Nat. Genet. 42:348. 10.1038/ng.548 20208533 PMC3092069

[B19] Karlsson B. Tsopelas P. Zamponi L. Capretti P. Soulioti N. Swedjemark G. (2008). Susceptibility to *Heterobasidion parviporum* in *Picea abies* clones grown in different environments. For. Pathol. 38, 83–89. 10.1111/j.1439-0329.2008.00543.x

[B20] Keriö S. Niemi S. M. Haapanen M. Daniel G. Asiegbu F. O. (2014). Infection of *Picea abies* clones with a homokaryotic isolate of *Heterobasidion parviporum* under field conditions. Can. J. For. Res. 45, 227–235. 10.1139/cjfr-2014-0247

[B21] Kolár T. Cermák P. Trnka M. Žid T. Rybníček M. (2017). Temporal changes in the climate sensitivity of Norway spruce and European beech along an elevation gradient in Central Europe. Agric. For. Meteorol. 239, 24–33. 10.1016/j.agrformet.2017.02.028

[B22] Kovalchuk A. Keriö S. Oghenekaro A. O. Jaber E. Raffaello T. Asiegbu F. O. (2013). Antimicrobial defenses and resistance in forest trees: challenges and perspectives in a genomic era. Annu. Rev. Phytopathol. 51, 221–244. 10.1146/annurev-phyto-082712-102307 23682916

[B23] Krokene P. Lahr E. Dalen L. S. Skrøppa T. Solheim H. (2012). Effect of phenology on susceptibility of Norway spruce (*Picea abies*) to fungal pathogens. Plant Pathol. 61, 57–62. 10.1111/j.1365-3059.2011.02487.x

[B24] Kump K. L. Bradbury P. J. Wisser R. J. Buckler E. S. Belcher A. R. Oropeza-Rosas M. A.. (2011). Genome-wide association study of quantitative resistance to southern leaf blight in the maize nested association mapping population. Nat. Genet. 43, 163–168. 10.1038/ng.747 21217757

[B25] Levkoev E. Kilpeläinen A. Luostarinen K. Pulkkinen P. Mehtätalo L. Ikonen V. (2017). Differences in growth and wood density in clones and provenance hybrid clones of Norway spruce. Can. J. For. Res. 47, 389–399. 10.1139/cjfr-2016-0285

[B26] Li H. Peng Z. Yang X. Wang W. Fu J. Wang J.. (2013). Genome-wide association study dissects the genetic architecture of oil biosynthesis in maize kernels. Nat. Genet. 45, 43–50. 10.1038/ng.2484 23242369

[B27] Lind M. Källman T. Chen J. Ma X. F. Bousquet J. Morgante M.. (2014). A *Picea abies* linkage map based on SNP markers identifies QTLS for four aspects of resistance to *Heterobasidion parviporum* infection. PLoS ONE 9:e101049. 10.1371/journal.pone.0101049 25036209 PMC4103950

[B28] Lindroth R. L. St. Clair S. B. (2013). Adaptations of quaking aspen (*Populus tremuloides* Michx.) for defense against herbivores. For. Ecol. Manage. 299, 14–21. 10.1016/j.foreco.2012.11.018

[B29] Luo M. Wang Y. Y. Liu X. Yang S. Lu Q. Cui Y.. (2012). HD2C interacts with HDA6 and is involved in ABA and salt stress response in *Arabidopsis*. J. Exp. Bot. 63, 3297–3306. 10.1093/jxb/ers059 22368268 PMC3350937

[B30] Luoranen J. Viiri H. (2016). Deep planting decreases risk of drought damage and increases growth of Norway spruce container seedlings. New For. 47, 701–714. 10.1007/s11056-016-9539-3

[B31] Monaghan J. Li X. (2010). The HEAT repeat protein ILITYHIA is required for plant immunity. Plant Cell Physiol. 51, 742–753. 10.1093/pcp/pcq038 20360018

[B32] Mukrimin M. Kovalchuk A. Neves L. G. Jaber E. Haapanen M. Kirst M. (2018). Data from: genome-wide exon-capture approach identifies genetic variants of Norway spruce genes associated with susceptibility to *Heterobasidion parviporum* infection. Dryad Digit. Repos. [Epub ahead of print]. 10.3389/fpls.2018.00793 PMC6005875 29946332

[B33] Müller H. Jimenez-Heredia R. Krolo A. Hirschmugl T. Dmytrus J. Boztug K.. (2017). VCF. Filter: interactive prioritization of disease-linked genetic variants from sequencing data. Nucleic Acids Res. 45, W567–W572. 10.1093/nar/gkx425 28520890 PMC5570181

[B34] Müller T. Freund F. Wildhagen H. Schmid K. J. (2015). Targeted re-sequencing of five Douglas-fir provenances reveals population structure and putative target genes of positive selection. Tree Genet. Genomes 11:816 10.1007/s11295-014-0816-z

[B35] Nemesio Gorriz M. Hammerbacher A. Ihrmark K. Källman T. Olson Å. Lascoux M.. (2016). Different alleles of a gene encoding leucoanthocyanidin reductase (PaLAR3) influence resistance against the fungus *Heterobasidion parviporum* in *Picea abies*. Plant Physiol. 171, 2671–2681. 10.1104/pp.16.00685 27317690 PMC4972290

[B36] Neves L. G. Davis J. M. Barbazuk W. B. Kirst M. (2013). Whole-exome targeted sequencing of the uncharacterized pine genome. Plant J. 75, 146–156. 10.1111/tpj.12193 23551702

[B37] Nystedt B. Street N. R. Wetterbom A. Zuccolo A. Lin Y. C. Scofield D. G.. (2013). The Norway spruce genome sequence and conifer genome evolution. Nature 497, 579–584. 10.1038/nature12211 23698360

[B38] Oliva J. Bernat M. Stenlid J. (2013). Heartwood stump colonisation by *Heterobasidion parviporum* and *H. annosum* s.s. in Norway spruce (*Picea abies*) stands. For. Ecol. Manage. 295, 1–10. 10.1016/j.foreco.2013.01.005

[B39] Pavy N. Gagnon F. Deschênes A. Boyle B. Beaulieu J. Bousquet J. (2016). Development of highly reliable *in silico* SNP resource and genotyping assay from exome capture and sequencing: an example from black spruce (*Picea mariana*). Mol. Ecol. Resour. 16, 588–598. 10.1111/1755-0998.12468 26391535

[B40] Piri T. Hamberg L. (2015). Persistence and infectivity of *Heterobasidion parviporum* in Norway spruce root residuals following stump harvesting. For. Ecol. Manage. 353, 49–58. 10.1016/j.foreco.2015.05.012

[B41] Ramírez V. López A. Mauch-Mani B. Gil M. J. Vera P. (2013). An extracellular subtilase switch for immune priming in Arabidopsis. PLoS Pathog. 9:e1003445. 10.1371/journal.ppat.1003445 23818851 PMC3688555

[B42] Robert J. A. Pitt C. Bonnett T. R. Yuen M. M. Keeling C. I. Bohlmann J.. (2013). Disentangling detoxification: Gene expression analysis of feeding mountain pine beetle illuminates molecular-level host chemical defense detoxification mechanisms. PLoS ONE 8:e77777. 10.1371/journal.pone.0077777 24223726 PMC3815198

[B43] Schaller A. Stintzi A. Rivas S. Serrano I. Chichkova N. V. Vartapetian A. B.. (2018). From structure to function – a family portrait of plant subtilases. New Phytol. 218, 901–915. 10.1111/nph.14582 28467631

[B44] Serrano I. Buscaill P. Audran C. Pouzet C. Jauneau A. Rivas S. (2016). A non canonical subtilase attenuates the transcriptional activation of defence responses in *Arabidopsis thaliana*. Elife 5:e19755. 10.7554/eLife.19755.001 27685353 PMC5074803

[B45] Skrøppa T. Solheim H. Hietala A. (2015). Variation in phloem resistance of Norway spruce clones and families to *Heterobasidion parviporum* and *Ceratocystis polonica* and its relationship to phenology and growth traits. Scand. J. For. Res. 30, 103–111. 10.1080/02827581.2014.963144

[B46] Sridha S. Wu K. (2006). Identification of *AtHD2C* as a novel regulator of abscisic acid responses in Arabidopsis. Plant J. 46, 124–133. 10.1111/j.1365-313X.2006.02678.x 16553900

[B47] Sun H. Paulin L. Alatalo E. Asiegbu F. O. (2011). Response of living tissues of *Pinus sylvestris* to the saprotrophic biocontrol fungus *Phlebiopsis gigantea*. Tree Physiol. 31, 438–451. 10.1093/treephys/tpr027 21551358

[B48] Swedjemark G. Stenlid J. (1995). Susceptibility of conifer and broadleaf seedlings to Swedish S and P strains of *Heterobasidion annosum*. Plant Pathol. 44, 73–79. 10.1111/j.1365-3059.1995.tb02717.x

[B49] Swedjemark G. Stenlid J. (1996). Variation in spread of *Heterobasidion annosum* in clones of *Picea abies* grown at different vegetation phases under greenhouse conditions. Scand. J. For. Res. 11, 137–144. 10.1080/02827589609382921

[B50] Swedjemark G. Stenlid J. Karlsson B. (2001). Variation in growth of *Heterobasidion annosum* among clones of *Picea abies* incubated for different periods of time. For. Pathol. 31, 163–175. 10.1046/j.1439-0329.2001.00238.x

[B51] Terhonen E. Marco T. Sun H. Jalkanen R. Kasanen R. Vuorinen M. (2011). The effect of latitude, season and needle-age on the mycota of scots pine (*Pinus sylvestris*) in Finland. Silva Fennica 45, 301–317. 10.14214/sf.104

[B52] Tian F. Bradbury P. J. Brown P. J. Hung H. Sun Q. Flint-Garcia S.. (2011). Genome-wide association study of leaf architecture in the maize nested association mapping population. Nat. Genet. 43, 159–162. 10.1038/ng.746 21217756

[B53] Tornero P. Mayda E. Gómez M. D. Cañas L. Conejero V. Vera P. (1996). Characterization of LRP, a leucine-rich repeat (LRR) protein from tomato plants that is processed during pathogenesis. Plant J. 10, 315–330. 10.1046/j.1365-313X.1996.10020315.x 8771787

[B54] Wang L. Gunulf A. Pukkala T. Rönnberg J. (2015). Simulated *Heterobasidion* disease development in *Picea abies* stands following precommercial thinning and the economic justification for control measures. Scand. J. For. Res. 30, 174–185. 10.1080/02827581.2014.978887

[B55] Wei X. Liu K. Zhang Y. Feng Q. Wang L. Zhao Y.. (2015). Genetic discovery for oil production and quality in sesame. Nat. Commun. 6:8609. 10.1038/ncomms9609 26477832 PMC4634326

[B57] Zubizarreta-Gerendiain A. Peltola H. Pulkkinen P. Kellomaki S. (2009). Effects of genetic entry and competition by neighbouring trees on growth and wood properties of cloned Norway spruce (*Picea abies*). Ann. For. Sci. 66:806 10.1051/forest/2009075

