# Supplementary material for: Genome-Wide Exon-Capture Approach Identifies Genetic Variants of Norway Spruce Genes Associated With Susceptibility to Heterobasidion parviporum Infection
Source: Front Plant Sci. 2018 Jun 12;9:793. doi: 10.3389/fpls.2018.00793 (PMC6005875; doi:10.3389/fpls.2018.00793)
Supplement: Supplementary file 1 [file Data_Sheet_1.DOCX]

**Supplementary file S1: Levene’s test for normality of the error variances and data distribution by skewness and kurtosis values**

| **Case Processing Summary** | | | | | | | |
| --- | --- | --- | --- | --- | --- | --- | --- |
|  | Tissues | Cases | | | | | |
|  |  | Valid | | Missing | | Total | |
|  |  | N | Percent | N | Percent | N | Percent |
| Lesion_size | Phloem_h | 341 | 100,0% | 0 | 0,0% | 341 | 100,0% |
|  | Phloem_v | 341 | 100,0% | 0 | 0,0% | 341 | 100,0% |
|  | Xylem_ho | 341 | 100,0% | 0 | 0,0% | 341 | 100,0% |
|  | Xylem_ve | 341 | 100,0% | 0 | 0,0% | 341 | 100,0% |

| **Descriptives** | | | | | |
| --- | --- | --- | --- | --- | --- |
|  | Tissues | | | Statistic | Std. Error |
| Lesion_size | Phloem_h | Mean | | 6,1408 | ,06820 |
|  |  | 95% Confidence Interval for Mean | Lower Bound | 6,0066 |  |
|  |  |  | Upper Bound | 6,2749 |  |
|  |  | 5% Trimmed Mean | | 6,1369 |  |
|  |  | Median | | 6,0000 |  |
|  |  | Variance | | 1,586 |  |
|  |  | Std. Deviation | | 1,25937 |  |
|  |  | Minimum | | 2,00 |  |
|  |  | Maximum | | 10,00 |  |
|  |  | Range | | 8,00 |  |
|  |  | Interquartile Range | | 2,00 |  |
|  |  | **Skewness** | | **,150** | **,132** |
|  |  | **Kurtosis** | | **1,320** | **,263** |
|  | Phloem_v | Mean | | 8,3255 | ,21159 |
|  |  | 95% Confidence Interval for Mean | Lower Bound | 7,9093 |  |
|  |  |  | Upper Bound | 8,7417 |  |
|  |  | 5% Trimmed Mean | | 7,8366 |  |
|  |  | Median | | 7,0000 |  |
|  |  | Variance | | 15,267 |  |
|  |  | Std. Deviation | | 3,90733 |  |
|  |  | Minimum | | 4,00 |  |
|  |  | Maximum | | 40,00 |  |
|  |  | Range | | 36,00 |  |
|  |  | Interquartile Range | | 3,00 |  |
|  |  | **Skewness** | | **3,552** | **,132** |
|  |  | **Kurtosis** | | **20,202** | **,263** |
|  | Xylem_ho | Mean | | 3,2287 | ,04338 |
|  |  | 95% Confidence Interval for Mean | Lower Bound | 3,1434 |  |
|  |  |  | Upper Bound | 3,3141 |  |
|  |  | 5% Trimmed Mean | | 3,1986 |  |
|  |  | Median | | 3,0000 |  |
|  |  | Variance | | ,642 |  |
|  |  | Std. Deviation | | ,80103 |  |
|  |  | Minimum | | 1,00 |  |
|  |  | Maximum | | 6,00 |  |
|  |  | Range | | 5,00 |  |
|  |  | Interquartile Range | | 1,00 |  |
|  |  | **Skewness** | | **,358** | **,132** |
|  |  | **Kurtosis** | | **,476** | **,263** |
|  | Xylem_ve | Mean | | 8,6188 | ,22617 |
|  |  | 95% Confidence Interval for Mean | Lower Bound | 8,1739 |  |
|  |  |  | Upper Bound | 9,0636 |  |
|  |  | 5% Trimmed Mean | | 8,1918 |  |
|  |  | Median | | 8,0000 |  |
|  |  | Variance | | 17,442 |  |
|  |  | Std. Deviation | | 4,17642 |  |
|  |  | Minimum | | 2,00 |  |
|  |  | Maximum | | 40,00 |  |
|  |  | Range | | 38,00 |  |
|  |  | Interquartile Range | | 4,00 |  |
|  |  | **Skewness** | | **2,814** | **,132** |
|  |  | **Kurtosis** | | **13,938** | **,263** |

| **Tests of Normality** | | | | | | | |
| --- | --- | --- | --- | --- | --- | --- | --- |
|  | Tissues | Kolmogorov-Smirnov^a^ | | | Shapiro-Wilk | | |
|  |  | Statistic | df | Sig. | Statistic | df | Sig. |
| Lesion_size | Phloem_h | ,187 | 341 | ,000 | ,925 | 341 | ,000 |
|  | Phloem_v | ,217 | 341 | ,000 | ,701 | 341 | ,000 |
|  | Xylem_ho | ,287 | 341 | ,000 | ,860 | 341 | ,000 |
|  | Xylem_ve | ,191 | 341 | ,000 | ,784 | 341 | ,000 |
| a. Lilliefors Significance Correction | | | | | | | |
