# Supplementary material for: Genome-Wide Exon-Capture Approach Identifies Genetic Variants of Norway Spruce Genes Associated With Susceptibility to Heterobasidion parviporum Infection
Source: Front Plant Sci. 2018 Jun 12;9:793. doi: 10.3389/fpls.2018.00793 (PMC6005875; doi:10.3389/fpls.2018.00793)

**Supplementary file S2: Levene’s test result for Histogram normal distribution, Q-Q plot, and Boxplot**

**Histograms**


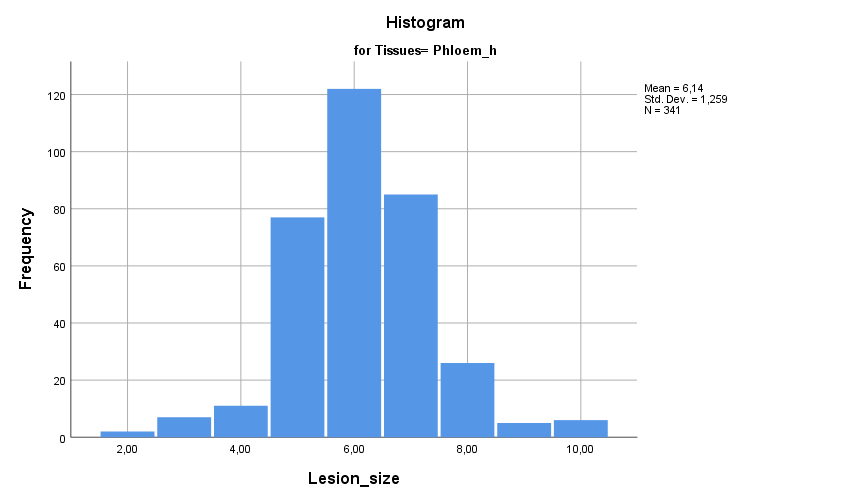

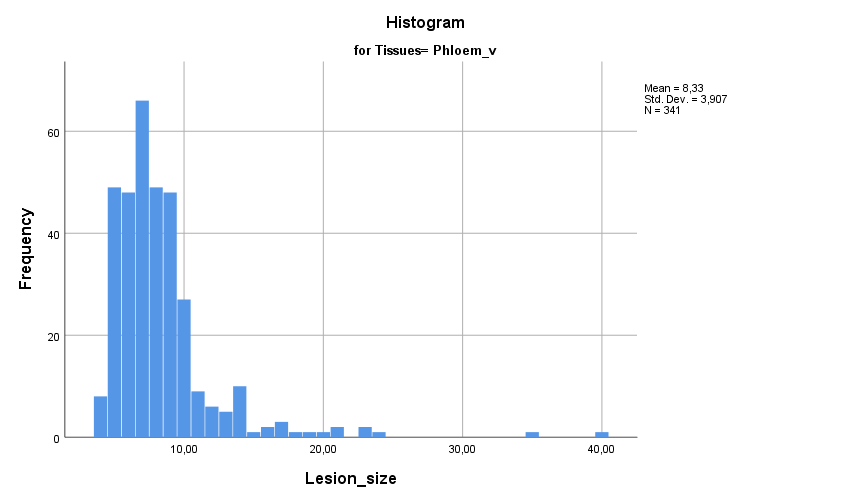


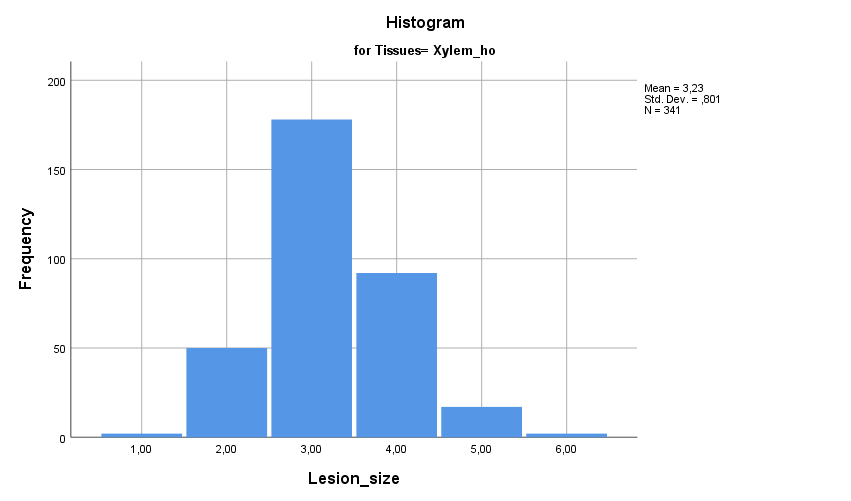

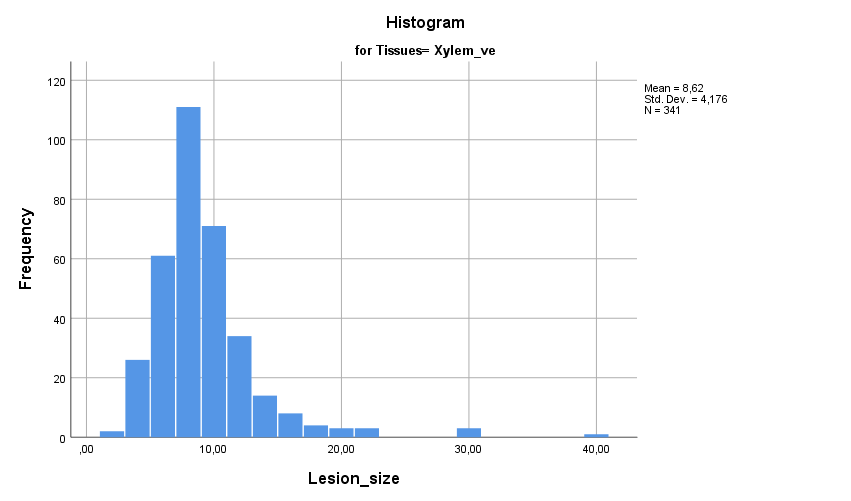


**Normal Q-Q Plots**


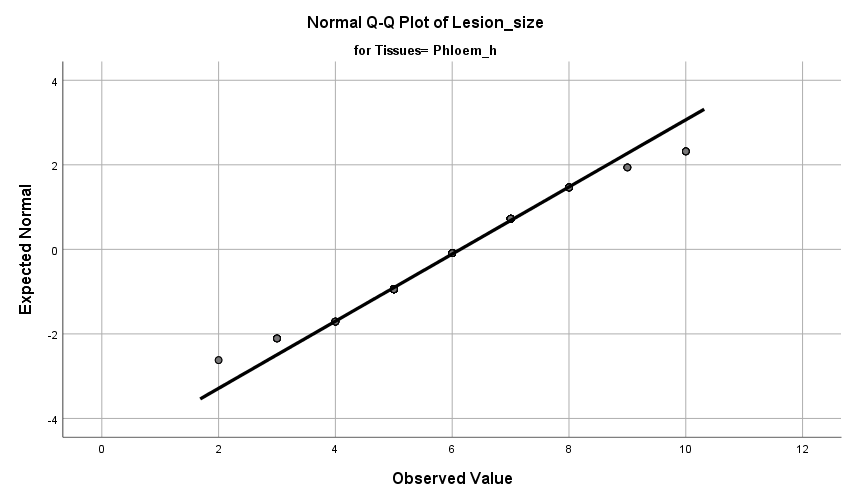


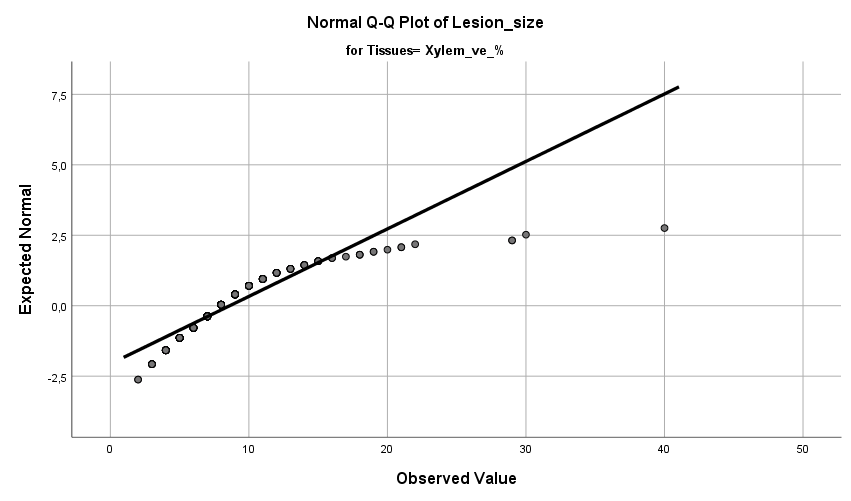


**Box plots**


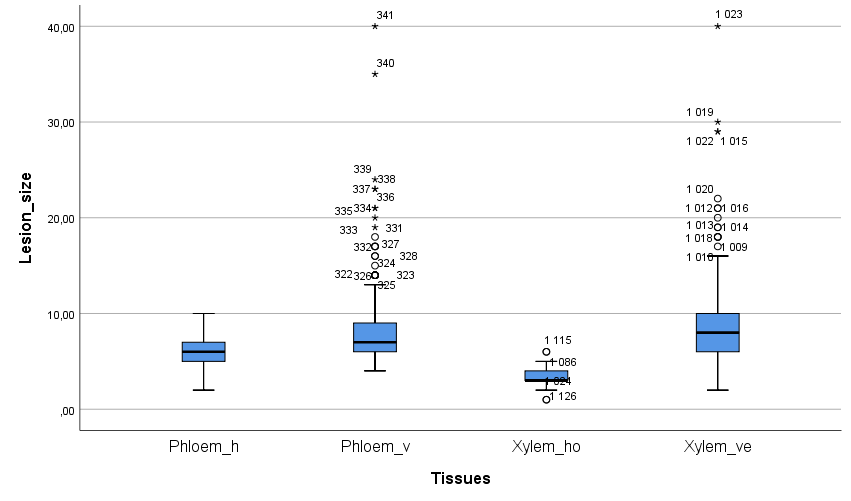

Supplement: Supplementary file 2 [file Data_Sheet_2.DOCX]
