# Supplementary material for: Genome-Wide Exon-Capture Approach Identifies Genetic Variants of Norway Spruce Genes Associated With Susceptibility to Heterobasidion parviporum Infection
Source: Front Plant Sci. 2018 Jun 12;9:793. doi: 10.3389/fpls.2018.00793 (PMC6005875; doi:10.3389/fpls.2018.00793)
Supplement: Supplementary file 3 [file Data_Sheet_3.DOCX]

Supplementary File S3. Q-Q and Manhattan plots for the single-marker associations. The red line represents 5% FDR and the x-axis for the Manhattan plot represent the SNP number, not chromosome location.

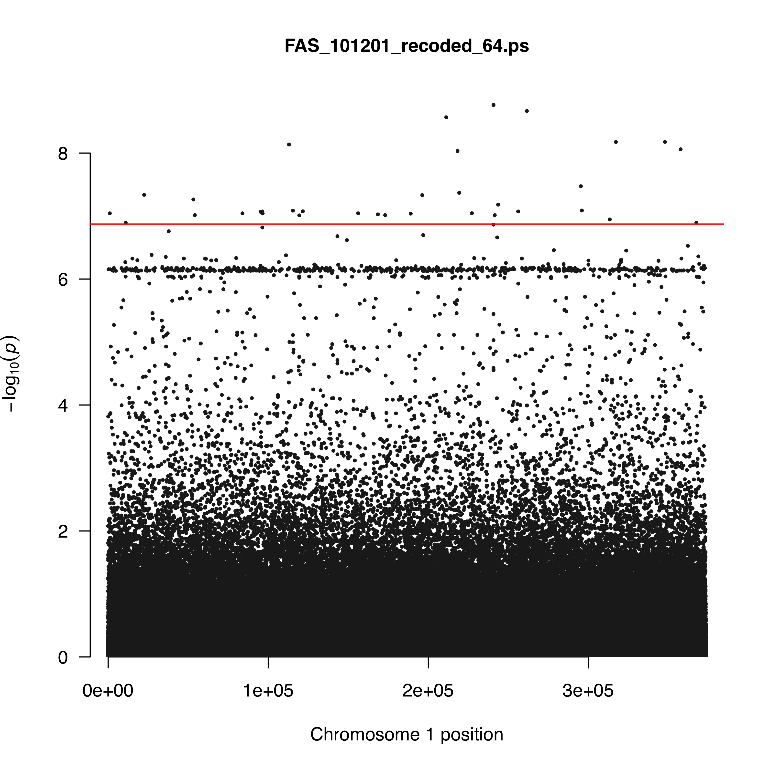


SNP number
