# Supplementary material for: Genome-Wide Exon-Capture Approach Identifies Genetic Variants of Norway Spruce Genes Associated With Susceptibility to Heterobasidion parviporum Infection
Source: Front Plant Sci. 2018 Jun 12;9:793. doi: 10.3389/fpls.2018.00793 (PMC6005875; doi:10.3389/fpls.2018.00793)
Supplement: Supplementary file 4 [file Data_Sheet_4.DOCX]

**Supplementary File S4.** SNPs identified by GWAS as significantly associated with the size of necrotic lesions formed in response to fungal inoculation.

| Contig | Pos | Beta | BONF | MAF | Gene model | Exon / intron | Predicted function | Nucleotide variant 0 | Nucleotide variant 1 | Effect |
| --- | --- | --- | --- | --- | --- | --- | --- | --- | --- | --- |
| MA_100379 | 20065 | -9.31 | 2.6E-03 | 0,016 | MA_100379g0010 | Intron | ATP synthase. D chain | C | T | n/a |
| MA_10253969 | 388 | -9.22 | 3.1E-03 | 0,016 | MA_10253969g0010 | Exon | similar to A. thaliana NF-YC11 (nuclear factor Y. subunit C11) | G | A | Arg (CGG) -> Gln (CAG) |
| MA_10426367 | 3936 | -7.68 | 3.9E-03 | 0,025 | MA_10426367g0010 | Intron | isopropylmalate dehydrogenase | C | G | n/a |
| MA_10430455 | 20871 | -5.22 | 1.3E-03 | 0,042 | MA_10430455g0010 | Intron | apolipoprotein | C | A | n/a |
| MA_10430576 | 10673 | -9.30 | 4.5E-03 | 0,017 | MA_10430576g0010 | Intron | anion-transporting ATPase | G | A | n/a |
| MA_10434266 | 9952 | -9.31 | 2.6E-03 | 0,016 | MA_10434266g0030 | Exon | disease resistance protein (TIR-NBS-LRR class) | T | C | Asn (AAU) -> Asp (GAU) (coding sequence is located on complementary strand) |
| MA_10435574 | 26634 | -5.00 | 1.7E-02 | 0,042 | MA_10435574g0010 | Intron | similar to A. thaliana SIGNAL PEPTIDE PEPTIDASE-LIKE 1 | A | T | n/a |
| MA_10435645 | 10536 | -9.32 | 2.0E-03 | 0,016 | MA_10435645g0010 | Exon | unchracterized hypothetical protein | C | T | Ser (UCC) -> Phe (UUC) |
| MA_10435655 | 10322 | -9.31 | 3.4E-03 | 0,016 | MA_10435655g0010 | Intron | uncharacterized hypothetical protein | C | G | n/a |
| MA_10436785 | 40331 | -10.46 | 1.1E-03 | 0,016 | MA_10436785g0010 | Intron | pentatricopeptide repeat (PPR) protein | C | A | n/a |
| MA_10436940 | 1868 | -5.85 | 4.3E-03 | 0,024 | MA_10436940g0010 | Exon | DnaJ chaperone protein | C | T | Thr (ACU) -> Ile (AUU) |
| MA_10437128 | 2574 | -9.30 | 4.6E-03 | 0,017 | MA_10437128g0010 | Intron | similar to A. thaliana MSI1 (MULTICOPY SUPRESSOR OF IRA1) | A | G | n/a |
| MA_10437232 | 6258 | -5.12 | 6.0E-03 | 0,042 | MA_10437232g0010 | Exon | uncharacterized hypothetical protein | G | C | no effect; Thr (ACC -> ACG) (coding sequence is located on complementary strand) |
| MA_10437232 | 6283 | -5.12 | 6.0E-03 | 0,042 | MA_10437232g0010 | Exon | uncharacterized hypothetical protein | C | A | Gly (GGG) -> Val (GUG) (coding sequence is located on complementary strand) |
| MA_136493 | 33555 | -9.31 | 2.6E-03 | 0,016 | MA_136493g0030 | Exon | uncharacterized hypothetical protein | G | A | Arg (CGG) -> Trp (UGG) (coding sequence is located on complementary strand) |
| MA_161300 | 32927 | -9.31 | 5.8E-03 | 0,025 | MA_161300g0010 | Intron | S-adenosyl-L-methionine-dependent methyltransferase | T | C | n/a |
| MA_171914 | 14464 | -9.30 | 4.5E-03 | 0,017 | MA_171914g0020 | Intron | ferric reduction oxidase | G | T | n/a |
| MA_19445 | 49416 | -9.32 | 5.6E-03 | 0,017 | MA_19445g0010 | Exon | Ribosomal protein L1p/L10e family | G | T | Gln (CAG) -> His (CAU) |
| MA_20554 | 10808 | -5.50 | 2.6E-02 | 0,047 | MA_20554g0010 | Intron | small nuclear ribonucleoprotein | G | A | n/a |
| MA_25943 | 3437 | -5.84 | 1.0E-04 | 0,035 | MA_25943g0010 | Intron | glutamate decarboxylase | A | T | n/a |
| MA_2908 | 3525 | -6.23 | 1.3E-04 | 0,024 | MA_2908g0010 | Exon | cellulose synthase | G | A | His (CAU) -> Tyr (UAU) |
| MA_296438 | 1075 | -5.50 | 3.0E-03 | 0,033 | MA_296438g0010 | Intron | phospholipase A2 | A | G | n/a |
| MA_330891 | 521 | -9.31 | 3.4E-03 | 0,016 | MA_330891g0010 | Intron | D-alanine D-alanine ligase family protein | G | C | n/a |
| MA_3905 | 29322 | -7.24 | 1.2E-05 | 0,032 | MA_3905g0010 | Intron | putative histone deacetylase | C | T | n/a |
| MA_3905 | 29323 | -7.24 | 1.2E-05 | 0,032 | MA_3905g0010 | Intron | putative histone deacetylase | T | G | n/a |
| MA_393542 | 3752 | -9.29 | 3.4E-03 | 0,016 | MA_393542g0020 | Exon | uncharacterized hypothetical protein | C | A | Arg (AGA) -> Ile (AUA) (coding sequence is located on complementary strand) |
| MA_4047 | 19725 | -4.74 | 2.1E-03 | 0,049 | MA_4047g0010 | Exon | subtilisin-like serine protease | T | C | no effect; Ser (AGU -> AGC) |
| MA_459865 | 4274 | -9.32 | 2.0E-03 | 0,016 | MA_459865g0020 | Exon | unchracterized hypothetical protein | C | A | Pro (CCU) -> His (CAU) |
| MA_479900 | 6145 | -5.48 | 6.9E-05 | 0,041 | MA_479900g0010 | Exon | uncharacterized F-box containing protein | G | A | Glu (GAG) -> Lys (AAG) |
| MA_6625107 | 548 | -6.05 | 2.7E-04 | 0,025 | MA_6625107g0010 | Exon | unchracterized hypothetical protein | G | T | Ala (GCA) -> Glu (GAA) (coding sequence is located on complementary strand) |
| MA_6661107 | 1408 | -5.85 | 4.2E-03 | 0,024 | MA_6661107g0010 | Exon | similar to A. thaliana CRUMBLED LEAF protein | C | T | Gly (GGC) -> Asp (GAC) (coding sequence is located on complementary strand) |
| MA_7662652 | 651 | -9.28 | 2.8E-03 | 0,018 | MA_7662652g0010 | Exon | disease resistance protein (TIR-NBS-LRR class) | C | T | Ser (UCA) -> Leu (UUA) |
| MA_78531 | 9949 | -10.47 | 6.0E-04 | 0,016 | MA_78531g0020 | Exon | unchracterized hypothetical (membrane?) protein | G | T | Pro (CCU) -> Thr (ACU) (coding sequence is located on complementary strand) |
| MA_9117754 | 6012 | -10.48 | 8.1E-04 | 0,016 | MA_9117754g0010 | Exon | unchracterized hypothetical protein | A | G | no effect; Asn (AAU -> AAC) (coding sequence is located on complementary strand) |
| MA_940838 | 2433 | -6.54 | 1.7E-04 | 0,042 | MA_940838g0010 | Exon | ILITHYIA. A. thaliana HEAT repeat protein involved in immunity | C | A | Ser (UCU) -> Tyr (UAU) |
| MA_97130 | 30350 | -9.22 | 3.1E-03 | 0,016 | MA_97130g0010 | Exon | ERF family transcription factor | G | A | no effect; Pro (CCG -> CCA) |
