# Supplementary material for: Genome-Wide Exon-Capture Approach Identifies Genetic Variants of Norway Spruce Genes Associated With Susceptibility to Heterobasidion parviporum Infection
Source: Front Plant Sci. 2018 Jun 12;9:793. doi: 10.3389/fpls.2018.00793 (PMC6005875; doi:10.3389/fpls.2018.00793)
Supplement: Supplementary file 5 [file Data_Sheet_5.DOCX]

**Supplementary File S5.** Sequence counts for the selected genes based on RNAseq analysis of five asymptomatic and five *Heterobasidion*-rotten Norway spruce trees.

| **Gene model** | **logFC** | **logCPM** | **Pvalue** | **S1** | **S2** | **S3** | **S4** | **S5** | **S6** | **S7** | **S8** | **S9** | **S10** |
| --- | --- | --- | --- | --- | --- | --- | --- | --- | --- | --- | --- | --- | --- |
| MA_100379g0010 | 0.10 | 4.78 | 0.77 | 635 | 501 | 514 | 441 | 358 | 691 | 513 | 545 | 593 | 281 |
| MA_10253969g0010 |  |  |  | n.d. | n.d. | n.d. | n.d. | n.d. | n.d. | n.d. | n.d. | n.d. | n.d. |
| MA_10426367g0010 | 0.31 | 3.51 | 0.35 | 196 | 165 | 243 | 149 | 184 | 272 | 201 | 273 | 215 | 198 |
| MA_10430455g0010 | 0.19 | 2.88 | 0.59 | 152 | 146 | 83 | 134 | 112 | 122 | 175 | 152 | 167 | 101 |
| MA_10430576g0010 | -0.03 | 3.28 | 0.94 | 206 | 223 | 175 | 134 | 162 | 185 | 173 | 197 | 187 | 142 |
| MA_10434266g0030 | -0.35 | 5.96 | 0.47 | 580 | 1587 | 844 | 580 | 2896 | 1965 | 544 | 1310 | 510 | 745 |
| MA_10435574g0010 | -0.09 | 5.27 | 0.76 | 793 | 812 | 623 | 701 | 751 | 674 | 718 | 666 | 595 | 801 |
| MA_10435645g0010 |  |  |  | n.d. | n.d. | n.d. | n.d. | n.d. | n.d. | n.d. | n.d. | n.d. | n.d. |
| MA_10435655g0010 | -0.43 | 3.85 | 0.18 | 396 | 342 | 263 | 295 | 225 | 262 | 225 | 225 | 219 | 197 |
| MA_10436785g0010 | 0.14 | 1.57 | 0.75 | 53 | 53 | 47 | 36 | 66 | 58 | 86 | 26 | 66 | 45 |
| MA_10436940g0010 | 0.65 | 0.75 | 0.11 | 34 | 16 | 20 | 26 | 18 | 54 | 28 | 30 | 32 | 36 |
| MA_10437128g0010 | 0.07 | 2.96 | 0.83 | 119 | 113 | 148 | 201 | 113 | 156 | 168 | 131 | 137 | 139 |
| MA_10437232g0010 | -0.27 | 3.88 | 0.49 | 297 | 336 | 469 | 231 | 141 | 381 | 209 | 282 | 183 | 173 |
| MA_10437232g0010 | -0.27 | 3.88 | 0.49 | 297 | 336 | 469 | 231 | 141 | 381 | 209 | 282 | 183 | 173 |
| MA_136493g0030 |  |  |  | n.d. | n.d. | n.d. | n.d. | n.d. | n.d. | n.d. | n.d. | n.d. | n.d. |
| MA_161300g0010 |  |  |  | n.d. | n.d. | n.d. | n.d. | n.d. | n.d. | n.d. | n.d. | n.d. | n.d. |
| MA_171914g0020 | 0.21 | 1.83 | 0.61 | 46 | 57 | 63 | 69 | 59 | 53 | 55 | 81 | 39 | 110 |
| MA_19445g0010 | -0.13 | 2.26 | 0.72 | 104 | 113 | 89 | 85 | 61 | 78 | 72 | 94 | 93 | 77 |
| MA_20554g0010 | 0.06 | 4.16 | 0.86 | 349 | 298 | 296 | 423 | 244 | 399 | 346 | 260 | 371 | 302 |
| MA_25943g0010 | 0.14 | 1.64 | 0.75 | 43 | 33 | 46 | 63 | 81 | 50 | 51 | 39 | 63 | 90 |
| MA_2908g0010 | -0.23 | 1.91 | 0.57 | 120 | 68 | 84 | 50 | 42 | 51 | 67 | 65 | 39 | 87 |
| MA_296438g0010 |  |  |  | n.d. | n.d. | n.d. | n.d. | n.d. | n.d. | n.d. | n.d. | n.d. | n.d. |
| MA_330891g0010 | 0.92 | 2.21 | 0.01 | 62 | 63 | 75 | 33 | 58 | 102 | 133 | 111 | 119 | 85 |
| MA_3905g0010 | 0.06 | 3.34 | 0.85 | 176 | 176 | 172 | 214 | 166 | 176 | 177 | 194 | 137 | 261 |
| MA_3905g0010 | 0.06 | 3.34 | 0.85 | 176 | 176 | 172 | 214 | 166 | 176 | 177 | 194 | 137 | 261 |
| MA_393542g0020 |  |  |  | n.d. | n.d. | n.d. | n.d. | n.d. | n.d. | n.d. | n.d. | n.d. | n.d. |
| MA_4047g0010 | 0.75 | 5.22 | 0.06 | 289 | 392 | 345 | 898 | 636 | 671 | 1118 | 358 | 1085 | 1078 |
| MA_459865g0020 |  |  |  | n.d. | n.d. | n.d. | n.d. | n.d. | n.d. | n.d. | n.d. | n.d. | n.d. |
| MA_479900g0010 | 0.11 | 2.97 | 0.76 | 132 | 133 | 122 | 192 | 108 | 141 | 192 | 97 | 212 | 101 |
| MA_6625107g0010 |  |  |  | n.d. | n.d. | n.d. | n.d. | n.d. | n.d. | n.d. | n.d. | n.d. | n.d. |
| MA_6661107g0010 | 0.00 | 0.34 | 1.00 | 19 | 11 | 23 | 23 | 31 | 20 | 18 | 17 | 29 | 24 |
| MA_7662652g0010 | 0.29 | 0.45 | 0.70 | 22 | 14 | 13 | 51 | 0 | 6 | 38 | 4 | 23 | 51 |
| MA_78531g0020 |  |  |  | n.d. | n.d. | n.d. | n.d. | n.d. | n.d. | n.d. | n.d. | n.d. | n.d. |
| MA_9117754g0010 | -0.48 | 2.12 | 0.23 | 76 | 135 | 105 | 64 | 80 | 105 | 58 | 66 | 66 | 38 |
| MA_940838g0010 | 0.23 | 4.04 | 0.45 | 278 | 280 | 301 | 292 | 236 | 275 | 306 | 403 | 286 | 361 |
| MA_97130g0010 | 0.97 | 4.63 | 0.01 | 291 | 263 | 162 | 529 | 304 | 603 | 861 | 364 | 687 | 514 |
